# Treatment patterns and survival in an exhaustive French cohort of pazopanib-eligible patients with metastatic soft tissue sarcoma (STS)

**DOI:** 10.1186/s12885-017-3057-3

**Published:** 2017-02-07

**Authors:** Isabelle Ray-Coquard, Olivier Collard, Françoise Ducimetiere, Mathieu Laramas, Florence Mercier, Nadine Ladarre, Stephanie Manson, Bertrand Tehard, Sébastien Clippe, Jean-Philippe Suchaud, Laetitia Stefani, Jean-Yves Blay

**Affiliations:** 1 Centre Léon-Bérard, University Claude Bernard Lyon I, 28 rue Laennec, 69008 Lyon, France; 2 Institut de Cancérologie de la Loire Lucien Neuwirth, 108 Bis av. Albert Raimond, 42270 Saint-Priest en Jarez, France; 3 CHU de Grenoble, Avenue Maquis du Grésivaudan, 38700 La Tronche, France; 4 Stat Process, 52 Boulevard Sébastopol, 75003 Paris, France; 5 Novartis, 2-4, rue Lionel Terray, Boite postale 308, F-92506 Rueil-Malmaison Cedex, France; 6 Novartis, Park View, Riverside Way, Watchmoor Park, Camberley, Surrey GU15 3YL UK; 7 Centre Marie Curie, 137 Avenue de Romans, 26000 Valence, France; 8 Service de Radiothérapie, Centre Hospitalier de Roanne, 28 Rue de Charlieu, 42300 Roanne, France; 9 Centre Hospitalier Annecy-Genevois, 1 Avenue de l’Hôpital, 74370 Metz-Tessy, France; 10 Service D’oncologie Médicale, Centre Léon Bérard, 28, rue Laennec, 69008 Lyon, France

**Keywords:** Sarcoma, Treatment, Survival, Metastatic, Chemotherapy, Database

## Abstract

**Background:**

The French EMS study prospectively collected exhaustive data from STS patients diagnosed in the Rhone-Alpes region from 2005 to 07.

**Methods:**

The database included diagnosis/histology, surgery, radiotherapy, systemic treatments and treatment response. Treatment patterns and outcomes of patients with metastatic disease, excluding adipocytic sarcoma and GIST were analyzed.

**Results:**

Of 888 total patients, 145 were included based on having metastatic disease and appropriate subtypes. All patients received treatment with systemic therapy being most common (74%, *n* = 107), followed by radiotherapy (30%, *n* = 44) and surgery (23%, *n* = 33). Doxorubicin, alone or in combination, was the most common first line systemic therapy (65%, *n* = 46). Drugs without license in sarcoma were used in 38–83% of treatments depending on treatment line. 24% of frontline patients demonstrated an objective response, decreasing to 11% objective responses in second line but no responses were documented beyond second line, with median PFS declining with each additional line. Median PFS also declined in patients receiving surgery compared to those receiving no surgery (8–15 m vs 5 m). Median OS from metastatic diagnosis for patients receiving systemic therapy was double that of patients without systemic treatment (24 m vs 12 m, *p* = 0.007).

**Conclusions:**

Outcomes in this population were poor and declined with successive treatment. However, results suggest that further anticancer therapies in recurrent sarcoma might be beneficial.

**Electronic supplementary material:**

The online version of this article (doi:10.1186/s12885-017-3057-3) contains supplementary material, which is available to authorized users.

## Background

Soft tissue sarcomas (STS) are rare malignant tumours, accounting for less than one percent of malignant neoplasms [1]. In France, the incidence of STS is estimated at 6 per 100,000 per year with 4000 new cases diagnosed each year [2]. With more than 50 distinct histological subtypes described [3–5], management of STS is challenging due to its rarity and clinical heterogeneity. Consequently, non-concordance in diagnosis occurs in 30% of cases [6], potentially resulting in delayed or inappropriate treatment.

Half of all STS patients with intermediate or high-grade tumors develop metastases [7]. Median survival is approximately 12 to 18 months from the time of metastatic diagnosis [8, 9], and has changed little in the past two decades. Five-year survival is no more than 8% in metastatic patients [8].

Chemotherapy is based on first-line anthracyclines, most commonly doxorubicin [10], providing objective responses in 12–26% of patients [11, 12]. The recent development of targeted therapies such as the tyrosine kinase inhibitor pazopanib [13] has led to hopes that the therapeutic prospects of patients with metastatic STS may be improved. Pazopanib demonstrated efficacy in a randomised, double-blind, placebo-controlled Phase III trial (PALETTE) in patients with metastatic STS (except adipocytic sarcoma) who received prior chemotherapy [14]. However, there are limited observational data on real-world treatment of patients with metastatic STS [15–17].

This study’s primary objective was to describe treatment patterns of patients with metastatic STS for whom pazopanib is labelled in a prospective registry of patients with a primary diagnosis of sarcoma. Secondary objectives were to determine survival rates and benefit of systemic treatment on survival.

## Methods

The EMS (Evaluation Médicale & Sarcome or Medical Evaluation and Sarcoma) study was an observational, prospective survey of patients with sarcoma from an exhaustive patient cohort in the Rhône-Alpes region of France over a 2-year period from March 2005 to March 2007. This sub-analysis considered all incident cases of metastatic soft tissue sarcoma (STS) identified in the database, corresponding to the subtypes eligible for the PALETTE study. Data was collected from patient records.

### EMS patient database

The EMS study was initiated as a comprehensive prospective population-based cohort in Rhône-Alpes in 2005, including STS, GIST and osteosarcoma. The primary referral centres for inclusion in the database are the 43 pathology laboratories in this region, which accounts for 10% of the French population. All cases underwent central histological review [18]. Methods for data collection in the EMS database have been described previously [18, 19].

### Patients

All patients with a diagnosis of primary sarcoma included in the EMS database between 1st March 2005 and 28th February 2007 with metastatic disease (initially or during follow up) were included in the present study.

The current analysis was restricted to patients with histological subtypes included in the PALETTE trial of pazopanib (i.e. key excluded subtypes: adipocytic sarcoma, osteosarcoma, gastrointestinal stromal tumour) [14].

### Data collection

Data in the EMS database included demographics, medical history, diagnostic procedures, primary tumour characteristics, functional status at diagnosis (ECOG score [20]), treatments (surgery, radiotherapy or chemotherapy) and outcomes according to the French 2006 SOR guidelines [20], follow-up recurrence and survival. It was noted whether patients were treated in an expert centre [21, 22] to describe the likely extent of collaboration between a multi-disciplinary specialist STS medical team. Data was prospectively extracted from the patient records covering the period from first inclusion into the EMS database (between March 2005 and March 2007) until a cut-off point of February 2012.

### Statistical analysis

Data presentation is principally descriptive. Quantitative data are presented as mean values ± standard deviation or median values [range] and categorical data as frequency counts and percentages. Overall survival, progression-free survival and time to recurrence were analysed using time-to-event analysis to generate Kaplan-Meier estimates with 95% confidence intervals. Missing data were not replaced.

## Results

### Patients

A total of 888 patients with a primary diagnosis of sarcoma were available for analysis. Among these 888 patients, 358 (40%) adult patients (>18 years of age) corresponded to the PALETTE trial’s eligibility criteria based on tumour subtype (Additional file 1: Table S1). The mean age at diagnosis was 61.5 ± 16.6 years and 47.2% of patients (169/358) were male. Of these 358 patients, 289 (80.7%) presented with a localised tumour and 69 (19.3%) presented with metastatic disease at diagnosis. A further 76 (21.2%) patients with initially localised disease progressed to metastatic disease during the observation period (2005–2012). The present analysis focuses on these 145 patients with metastatic disease.

In the 145 patients with metastatic disease, 57% were men with a mean age at diagnosis of 60 years for those initially diagnosed with metastatic disease and 63 for those diagnosed with local disease who went on to develop metastases. The initial tumour site was visceral for 58% of patients, with a median time to progression of 12 m (95%CI 7-16 m) for those with initially local disease. Metastases were present in a single distant site in 93 patients (64.1%), in two distant sites in 37 patients (25.5%) and in three or more distant sites in the remaining 15 patients (10.3%). The principal metastatic sites were the lung (103 patients; 71.0%), liver (35 patients; 24.1%), and bone (15 patients; 10.3%).

### Treatment of metastatic sarcoma

All patients with metastases received at least one line of treatment (surgery +/− radiotherapy +/− systemic treatment), 83 patients (57.5%) received two treatment lines, 49 (33.8%) three lines and 29 (20.0%) four or more lines. Treatments provided by line of therapy are presented in Table 1. The first treatment line was principally systemic treatment in more than two-thirds of patients (68.8%). Surgery or radiotherapy for metastatic disease were used in less than twenty percent of patients. Treatment across modalities ((Metastatic Surgery (MS) +/− Radiotherapy (RT) +/− Systemic treatment (ST)) was only received by 25 patients (22.5%) in 1st treatment line, 14 patients (18.9%) in 2nd line and 1 patient (2.4%) in 3rd line. The most frequent combinations were MS + ST (10%) and RT + ST (14%).

**Table 1:** Treatment of metastatic sarcoma by treatment line

	Any treatment line	1st treatment line	2nd treatment line	3rd treatment line	4th or higher treatment line
	*N* = 145	*N* = 145	*N* = 83	*N* = 49	*N* = 52^a^
Metastatic Surgery (MS)	33 (22.8%)	25 (19.1%)	11 (14.5%)	1 (2.2%)	2 (3.9%)
Missing data		14	7	4	1
Radiotherapy (RT)	44 (30.3%)	20 (14.5%)	16 (20.5%)	6 (13.0%)	11 (22.0%)
Missing data		7	5	3	2
Systemic treatment (ST)	107 (73.8%)	95 (68.8%)	62 (78.5%)	35 (72.9%)	42 (82.4%)
Missing data		7	4	1	1
Treatment combinations	*N* = 145	*N* = 111	*N* = 74	*N* = 41	-
MS + RT	2 (1.4%)	1 (0.9%)	1 (1.4%)	0 (0.0%)	-
MS + ST	14 (9.7%)	11 (9.9%)	4 (5.4%)	0 (0.0%)	-
RT + ST	20 (13.8%)	9 (8.1%)	8 (10.2%)	1 (2.4%)	-
MS + RT + ST	5 (3.4%)	4 (3.6%)	1 (1.4%)	0 (0.0%)	-

*MD* missing data are related to incomplete information regarding the specific types of treatment given

^a^as all treatment lines ≥ 4 were counted, the same individual patient may have been counted twice or more if they received multiple lines of treatment

Systemic treatments are described in Table 2. Among the patients treated with a 1st line of systemic treatment, one third (*n* = 36) received an experimental therapy in the context of a clinical trial. In 2nd and 3rd line, around 27% of patients entered a clinical trial. Among the 71 patients receiving systemic treatment, 34 different chemotherapeutic regimens were used.

**Table 2:** Systemic treatment of metastatic sarcoma by line

	1st systemic treatment line	2nd systemic treatment line	3rd systemic treatment line	4th systemic treatment line
	*N* = 107	*N* = 59	*N* = 34^d^	*N* = 18
Clinical trial^a^				
Yes	36 (33.6%)	16 (27.1%)	9 (27.3%)	0 (0.0%)
No	71 (66.4%)	43 (72.9%)	24 (72.7%)	18 (100.0%)
Treatment class^a^				
Cytotoxic agent	65 (91.6%)^b^	37 (86.0%)^b^	22 (91.7%)^b^	12 (66.6%)^b^
Hormonal therapy	2 (2.8%)	1 (2.3%)	0 (0.0%)	0 (2.4%)
Targeted therapy	4 (5.6%)	5 (11.7%)	2 (8.3%)	6 (33.4%)
Treatment combinations^a^				
Monotherapy	38 (53.5%)	30 (69.8%)	9 (37.5%)	17 (94.4%)
Combination	33 (46.5%)	13 (30.2%)	15 (62.5%)	1 (0.6%)
Individual agent^a,c^				
Doxorubicin	46 (64.7%)	10 (23.3%)	1 (4.2%)	0 (0.0%)
Ifosfamide	17 (23.9%)	6 (14.0%)	3 (12.5%)	1 (5.5%)
Dacarbazine	8 (11.3%)	1 (2.3%)	1 (4.2%)	0 (0.0%)
Gemcitabine	11 (15.5%)	11 (25.6%)	12 (50.0%)	2 (11.1%)
Docetaxel	11 (15.5%)	10 (23.3%)	10 (41.7%)	0 (0.0%)
Trabectedin	2 (2.8%)	9 (20.9%)	4 (16.4%)	7 (38.9%)
At least one off-label agent				
Yes	27 (38.0%)	20 (46.5%)	20 (83.3%)	11 (61.1%)
No	44 (62.0%)	23 (53.5%)	4 (16.3%)	7 (38.9%)

^a^percentages are calculated with respect to the total number of patients receiving chemotherapy

^b^Certain patients (3 at 1st line, & at 2nd line, 2 at 3rd line and 2 at 4th or higher line) received a combination of a cytotoxic agent and a targeted therapy, the classes of treatment used are therefore not mutually exclusive

^c^Only the most frequently used agents are listed; these drugs are frequently used in combinations, so the drugs are not mutually exclusive

^d^Therapy data missing on 1 patient

For first line systemic treatment, the most frequently used agent was doxorubicin in 46 patients (64.7%), either in monotherapy (27 patients) or in combination (19 patients). In second line, the most frequently used agent was gemcitabine, in 11 patients (25.6%), but there was no drug that most clearly dominated second line treatment or beyond. Drugs without a labelled indication for STS were used with increasing frequency in later lines of therapy.

### Care management via sarcoma network

Care management in localized or metastatic disease was usually initiated (91.0%) in centres outside the Netsarc/GSF-GETO network (Table 3). In patients whose early disease became metastatic, less than 7% of patients were fully managed in the GSF-GETO network. For patients with mSTS therapeutic decisions were partially/fully initiated by the GSF-GETO network for 46.9% (68/145) of patients. The GSF-GETO network was more involved in therapeutic decisions when metastatic STS was primarily diagnosed (36 patients; 52.2%).

**Table 3:** Care management via NETSARC/GSF-GETO network

		Local sarcoma with NO further metastatic lines	Local sarcoma WITH further metastatic lines	Sarcoma initially metastatic	Total
		*N* = 213	*N* = 76	*N* = 69	*N* = 358
Initial care by the NetSarc network	N	213	76	69	358
No	n (%)	196 (92.0%)	67 (88.2%)	62 (89.9%)	325 (90.8%)
Yes	n (%)	17 (8.0%)	9 (11.8%)	7 (10.1%)	33 (9.2%)
Full treatment course	N	213	76	69	358
Outside the GSF-GETO network	n (%)	136 (63.8%)	44 (57.9%)	33 (47.8%)	213 (59.5%)
Care by the GSF-GETO network only	n (%)	7 (3.3%)	5 (6.6%)	4 (5.8%)	16 (4.5%)
Mixed care	n (%)	70 (32.9%)	27 (35.5%)	32 (46.4%)	129 (36.0%)

### Clinical outcome

Clinical outcomes for patients with metastatic disease are presented in Tables 4 and 5 and Additional file 2: Table S2.

**Table 4:** Treatment outcomes by treatment line

	1st line	2nd line	3rd line	4th or higher line
	*N* = 145	*N* = 83	*N* = 49	*N* = 52^a^
Time to relapse (months; median [range])	12.1 [1.1–72.2]^b^	8.2 [1.2–37.9]	4.1 [0.5–27.7]	3.4 [0.7–23.2]
Treatment-free interval^c^	*N* = 46^b^	*N* = 62	*N* = 35	*N* = 42
(months; median [range])	10.2 [0.7–49.0]	2.9 [0.0–29.8]	1.0 [0.0–10.6]	1.0 [0.0–18.4]
Response at end of therapy	*N* = 89	*N* = 61	*N* = 33	*N* = 36
Complete response (CR)	3 (3.4%)	None	None	None
Partial response (PR)	19 (21.3%)	8 (13.1%)	None	None
Stable disease (SD)	19 (21.3%)	10 (16.4%)	4 (12.1%)	3 (8.3%)
Progressive disease	48 (54.0%)	43 (70.5%)	29 (87.9%)	33 (91.7%)
CP + PR	22 (24.7%)	8 (13.1%)	None	None
CP + PR + SD	41 (46.0%)	18 (29.5%)	4 (12.1%)	3 (8.3%)

^a^as all treatment lines ≥ 4 were counted, the same individual patient may have been counted multiple times with multiple treatment lines

^b^Data only for patients without metastases at diagnosis

^c^Time between end of the previous systemic treatment and start of current treatment line (for 1st line: duration from initial diagnosis to first systemic therapy)

**Table 5:** Treatment outcomes by systemic treatment line

	1st systemic treatment line	2nd systemic treatment line	3rd systemic treatment line	4th systemic treatment line
	*N* = 107 (MD = 38)	*N* = 59	*N* = 34	*N* = 18
Time to relapse (months; median [range])	10.6 [1.1–72.2]	8.0 [1.4–27.4]	3.7 [0.5–27.7]	6.8 [1.6–21.5]
Treatment-free interval^a^	*N* = 19	*N* = 58	*N* = 33	*N* = 18
(months; median [range])	10.7 [0.7–49.0]	2.8 [0.0–19.6]	1.4 [0.0–18.4]	1.0 [0.0–5.8]
Response at end of therapy	*N* = 102	*N* = 57	*N* = 32	*N* = 16
Complete response (CP)	3 (2.9%)	None	None	None
Partial response (PR)	21 (20.6%)	6 (10.5%)	None	None
Stable disease (SD)	22 (21.6%)	7 (12.3%)	5 (15.6%)	2 (12.5%)
Progressive disease	56 (54.9%)	44 (77.2%)	27 (84.4%)	14 (87.5%)
CP + PR	24 (23.5%)	6 (10.5%)	None	None
CP + PR + SD	46 (45.1%)	13 (22.8%)	5 (15.6%)	2 (12.5%)

^a^Time between the end of the previous systemic treatment and the start of the current treatment line

From the beginning of the first metastatic treatment, the median time to relapse was 12.1 months (range: 1.1-72.2), and this time decreased with each subsequent treatment line. Similarly, response rates decreased with treatment line, with no patients responding completely or partially after the end of the second treatment line.

**Fig. 1:**
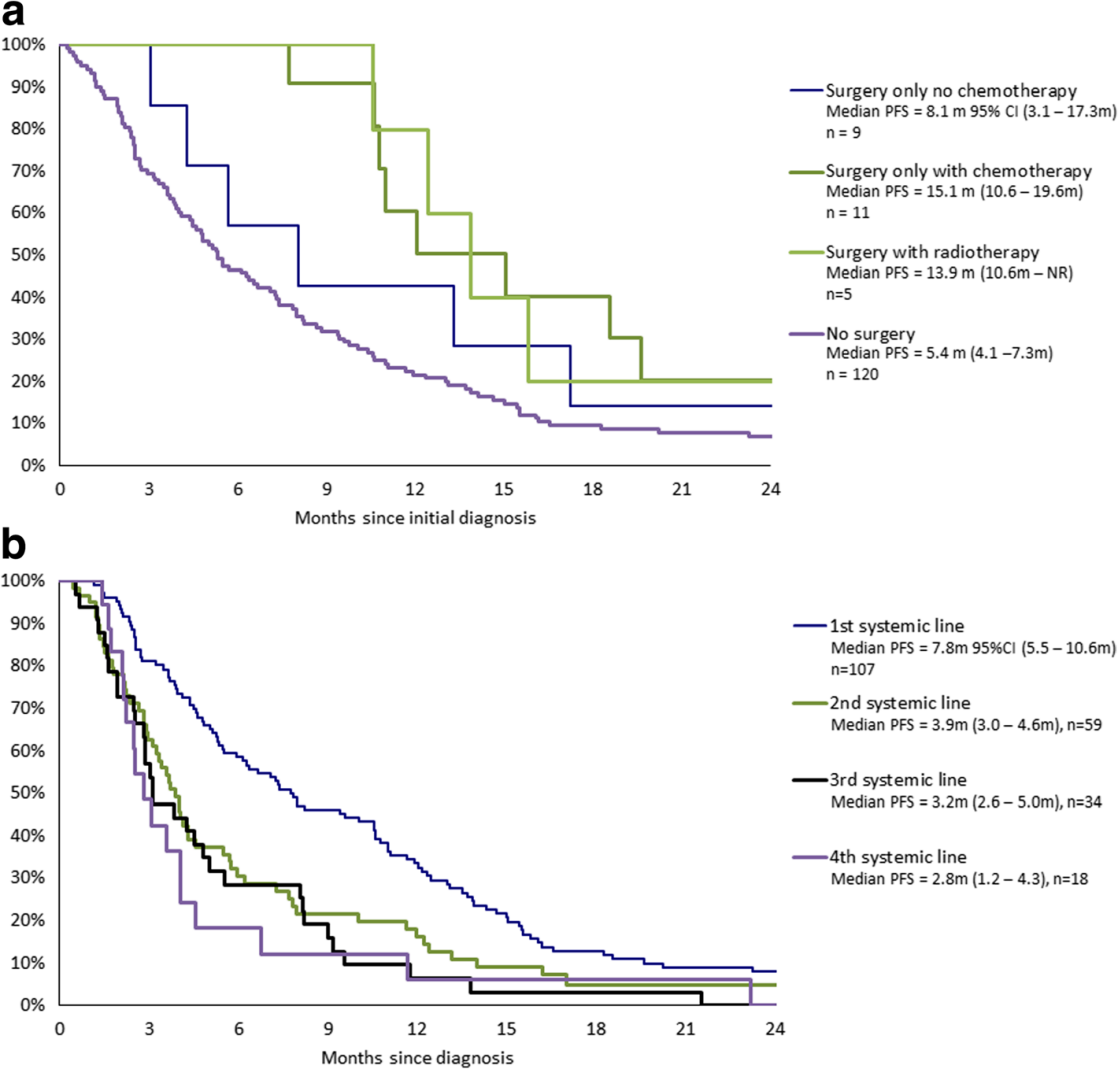
**a** Kaplan Meier PFS curves for patients with metastatic disease according to 1st line treatment options. **b** Kaplan Meier PFS curves for patients with metastatic disease according to systemic treatment lines

Progression free survival (PFS) was estimated (Fig. 1a) according to 1st metastatic treatment. Although only few patients received surgery for their metastatic disease, it appears that patients selected for surgery exhibited improved PFS but adjuvant treatment seems to do better than surgery alone for PFS.

A similar approach was undertaken considering only patients receiving systemic treatment (including *n* = 3 adjuvant/neoadjuvant systemic therapies). Similarly, the time to relapse and response rates decreased with each subsequent systemic treatment line (Tables 4 and 5, Fig. 1b). No patients responded completely or partially after the end of the second systemic treatment line.

The median overall survival from initial diagnosis with sarcoma was 20.2 months (95% CI 15.9–27.0 m).

**Fig. 2:**
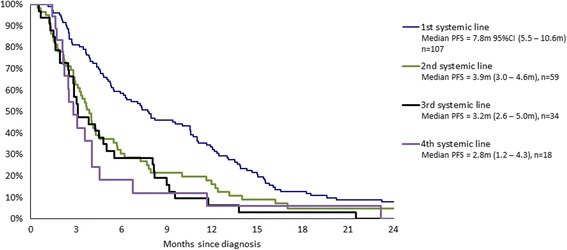
Kaplan Meier OS curves for patients with metastatic disease according to whether chemotherapy was received

Comparing the overall survival of patients receiving chemotherapy versus those who did not (Fig. 2), patients receiving chemotherapy had significantly longer overall survival (24.2 m [95% CI 17.4–33.6 m] vs. 11.8 m [95% CI 6.5–19.6 m] *p* = 0.007). No sub-analysis was done by performance status due to >50% missing data.

## Discussion

The present study reported treatment patterns and outcomes in patients with metastatic STS, excluding liposarcoma and GIST, enrolled in an exhaustive prospective patient database in real life.

Of the entire STS population, 40.5% (145/358) were diagnosed with or subsequently developed metastatic disease. 26.3% (76/289) of patients with localised STS had metastatic relapse, − half the rate of metastatic relapse reported in the literature [23]. Previous hospital case-series studies conducted in specialist centres may be enriched with more severe patients at higher risk for progression or relapse [17], whereas this study was population based including all incident cases. In this study, the treatment rate for metastatic STS in non-specialist centres was lower, around 53%.

Systemic chemotherapy was the most common first-line strategy for patients with metastatic STS with 68.8% of patients receiving at least one line.

A large variety of systemic therapies were prescribed. The European Society for Medical Oncology clinical practice guidelines for STS [24–26] have been updated regularly since 2007 and in 2014. They confirm that although there is no formal demonstration that multi-agent chemotherapy is superior to single agent doxorubicin therapy, multi-agent chemotherapy with adequate-dose anthracyclines plus ifosfamide may be the treatment of choice for advanced disease, particularly when a tumour response is felt to be potentially advantageous and patient performance status is good. During the EMS study enrolment period, doxorubicin was recommended alone or in association to other chemotherapies [25]. In this study, 64.7% of patients receiving a systemic therapy were prescribed doxorubicin, comparable to 70% previously documented in the SABINE study [15]. In addition, 38% of patients in the present study were prescribed unlicensed systemic agents. Approved treatment options were limited, with only anthracyclines, ifosfamide, dacarbazine and trabectedin being registered for use in sarcoma in France during this period. This may reflect physician perceptions of the inadequacy of approved treatments. A similar perception may underlie the relatively high proportion of patients who are included in clinical trials. A high percentage of patients (36%) were diagnosed with Sarcoma NOS, making it difficult to understand whether a tailored treatment might be impactful.

During subsequent treatment lines, no dominant treatment protocol was observed with doxorubicin remaining the most widely-used individual chemotherapeutic agent. Although ifosfamide or dacarbazine are recommended in ESMO guidelines for patients who fail to respond to first-line doxorubicin, these agents were not widely used. As in the SABINE study [15], the most widely used combination was gemcitabine with docetaxel, in spite of the fact that it is not registered for treatment of metastatic STS. Trabectedin was used more frequently in second and later treatment lines, consistent with the approved indication and ESMO guidelines. Targeted therapies were principally used in late treatment lines.

Clinical outcomes in this study population were poor and declined with each successive line of treatment. Median time to relapse after first-line systemic treatment was around one year, the treatment-free interval between first- and second- line was around ten months. Complete or partial response rates to first-line therapy were less than 25%, which is similar to response rates for anthracycline-based therapy published in the literature [11, 12] and no patients responded to third or higher line treatments. Median overall survival was twice as high in patients receiving chemotherapy versus those who didn’t. This should be interpreted with caution, as some patients might be too weak to be able to receive chemotherapy, have comorbidities, or otherwise might not be appropriate or wish to receive therapy, which could impact the comparison. However, it is illuminating to see a clear difference in OS between those receiving and not receiving chemotherapy (24 m vs 12 m, *p* = 0.007), and is consistent with observations in breast and ovarian cancer demonstrating chemotherapy being linked with longer survival [27, 28]. Similar selection bias combined with small patient numbers undergoing surgery similarly limit the interpretation of the difference in PFS according to surgery.

The heterogeneous approach to treatment and poor outcome observed here and elsewhere emphasises the need for better coordination of diagnosis and treatment. In 2010, the French Cancer Institute (INCa) identified a national network of regional Expert Centres for the management of STS (NETSARC), composed mostly of members of the French Sarcoma Group, called GSF-GETO (Groupe Sarcome Français – Groupe d’Etude des Tumeurs Osseuses). It is responsible for coordinating care between the NETSARC expert STS medical team and other oncologists in the territory in order to optimise patient care. GSF-GETO published STS best practice guidelines in 2006 [20], conformity to which have demonstrably improved PFS for sarcoma patients [29]. In this study, prior to NETSARC’s creation, the majority of patients were managed outside GSF-GETO centres both at initial diagnosis (91.0%) and during metastatic disease (53.1%). There is no doubt that the NETSARC network will improve the management of STS.

The study’s major strengths is the exhaustive coverage using a patient registry, across a large French region, the prospective design allowing complete documentation of the disease, and the naturalistic setting in routine clinical practice in France. This analysis was limited to patients with STS with relapsing disease with subtypes included in the PALETTE study.

## Conclusion

In conclusion, this study demonstrates that treatment of metastatic STS in everyday practice in Rhone-Alpes region is highly heterogeneous and associated with poor outcomes. This highlights the significant unmet medical needs with respect to standardised treatment protocols and more effective therapies.

## Additional files

Additional file 1: Table S1. Characteristics of included sarcomas. (DOCX 15 kb)
Additional file 2: Table S2. Cause of death. (DOCX 35 kb)

## Abbreviations

CI: Confidence interval
EMS: Evaluation Médicale et Sarcome or Medical Evaluation and Sarcoma
ESMO: European Society for Medical Oncology
GIST: Gastrointestinal stromal tumor
GSF-GETO: Groupe Sarcome Français – Groupe d’Etude des Tumeurs Osseuses
MS: Metastatic surgery
NETSARC: Network of regional Expert Centres for the management of STS
OS: Overall survival
PFS: Progression free survival
RT: Radiotherapy
ST: Systemic treatment
STS: Soft tissue sarcoma
