# Supplementary material for: Treatment patterns and survival in an exhaustive French cohort of pazopanib-eligible patients with metastatic soft tissue sarcoma (STS)
Source: BMC Cancer. 2017 Feb 7;17:111. doi: 10.1186/s12885-017-3057-3 (PMC5297166; doi:10.1186/s12885-017-3057-3)
Supplement: Additional file 1: Table S1. — Characteristics of included sarcomas. (DOCX 15 kb) [file 12885_2017_3057_MOESM1_ESM.docx]

**Table S1.** Characteristics of included sarcomas.

| **Standardised diagnosis** | **Local Sarcoma at Diagnosis** | | **Metastatic Sarcoma at Diagnosis**  N=69 | **All Patients**  N=358 |
| --- | --- | --- | --- | --- |
|  | No further metastatic disease  N=213 | Further metastatic disease  N=76 |  |  |
| Sarcoma NOS | 69 (32.4%) | 32 (42.1%) | 26 (37.7%) | 127 (35.5%) |
| Leiomyosarcoma | 42 (19.7%) | 10 (13.2%) | 16 (23.2%) | 68 (19.0%) |
| Angiosarcoma | 12 (5.6%) | 5 (6.6%) | 9 (13.0%) | 26 (7.3%) |
| Uterine leiomyosarcoma | 8 (3.8%) | 10 (13.2%) | 7 (10.1%) | 25 (7.0%) |
| Myxofibrosarcoma | 19 (8.9%) | 3 (3.9%) | 0 (0.0%) | 22 (6.1%) |
| Endometrial stromal sarcoma | 14 (6.6%) | 1 (1.3%) | 2 (2.9%) | 17 (4.7%) |
| Synovial sarcoma | 10 (4.7%) | 5 (6.6%) | 0 (0.0%) | 15 (4.2%) |
| Malignant peripheral nerve sheath tumour | 11 (5.2%) | 0 (0.0%) | 0 (0.0%) | 11 (3.1%) |
| Solitary malignant fibrous tumour | 6 (2.8%) | 2 (2.6%) | 1 (1.4%) | 9 (2.5%) |
| Low grade fibromyxoid sarcoma | 6 (2.8%) | 0 (0.0%) | 0 (0.0%) | 6 (1.7%) |
| Epithelioid sarcoma | 4 (1.9%) | 0 (0.0%) | 1 (1.4%) | 5 (1.4%) |
| Fusiform cell sarcoma | 2 (0.9%) | 2 (2.6%) | 1 (1.4%) | 5 (1.4%) |
| Others | 10 (4.7%) | 6 (7.9%) | 6 (8.7%) | 22 (6.1%) |
