# Supplementary material for: Treatment patterns and survival in an exhaustive French cohort of pazopanib-eligible patients with metastatic soft tissue sarcoma (STS)
Source: BMC Cancer. 2017 Feb 7;17:111. doi: 10.1186/s12885-017-3057-3 (PMC5297166; doi:10.1186/s12885-017-3057-3)
Supplement: Additional file 2: Table S2. — Cause of death. (DOCX 35 kb) [file 12885_2017_3057_MOESM2_ESM.docx]

Table S2. Cause of death

| **Survival Status** | **Dead**  **N=120** |
| --- | --- |
| Follow-up duration (months) Median [IQR] | 15.9 [7.4-32.5] |
| Cause of death  Death due to cancer  Death due to 2^nd^ cancer  Therapeutic complications  Other*(details)  Death with no other information | 114 (95.0%) 1 (0.8%) 1 (0.8%) 3 (2.5%)  1 (0.8%) |
